# Supplementary material for: The human TRPA1 intrinsic cold and heat sensitivity involves separate channel structures beyond the N-ARD domain
Source: Nat Commun. 2022 Oct 17;13:6113. doi: 10.1038/s41467-022-33876-8 (PMC9576766; doi:10.1038/s41467-022-33876-8)
Supplement: Supplementary file 1 — Supplementary Information [file 41467_2022_33876_MOESM1_ESM.pdf]

Supplementary Information

The human TRPA1 intrinsic cold and heat sensitivity involves separate channel structures beyond the N-ARD domain

Lavanya Moparthi[1,2*], Viktor Sinica[3], Vamsi K Moparthi[4], Mohamed Kreir[5], Thibaut Vignane[6], Milos Filipovic[6], Viktorie Vlachova[3] and Peter M. Zygmunt[7*]

[1]Wallenberg Centre for Molecular Medicine, Linköping University, SE-581 83 Linköping, Sweden; and [2]Department of Biomedical and Clinical Sciences (BKV), Faculty of Health Sciences, Linköping University, SE-581 83 Linköping, Sweden. [3]Department of Cellular Neurophysiology, Institute of Physiology of the Czech Academy of Sciences, 142 20 Prague, Czech Republic. [4]Department of Physics, Chemistry, and Biology, Division of Chemistry, Linköping University, SE-58183 Linköping, Sweden. [5]Janssen Research & Development, Division of Janssen Pharmaceutica N.V., Turnhoutseweg 30, 2340 Beerse, Belgium. [6]Leibniz-Institut für Analytische Wissenschaften-ISAS-e.V. Bunsen-Kirchhoff-Straße 11, 44139 Dortmund, Germany. [7]Department of Clinical Sciences Malmö, Lund University, SE-214 28 Malmö, Sweden.

*Correspondence: lavanya.moparthi@liu.se or peter.zygmunt@med.lu.se

Supplementary Figures 1-4
Supplementary Tables 1 and 2

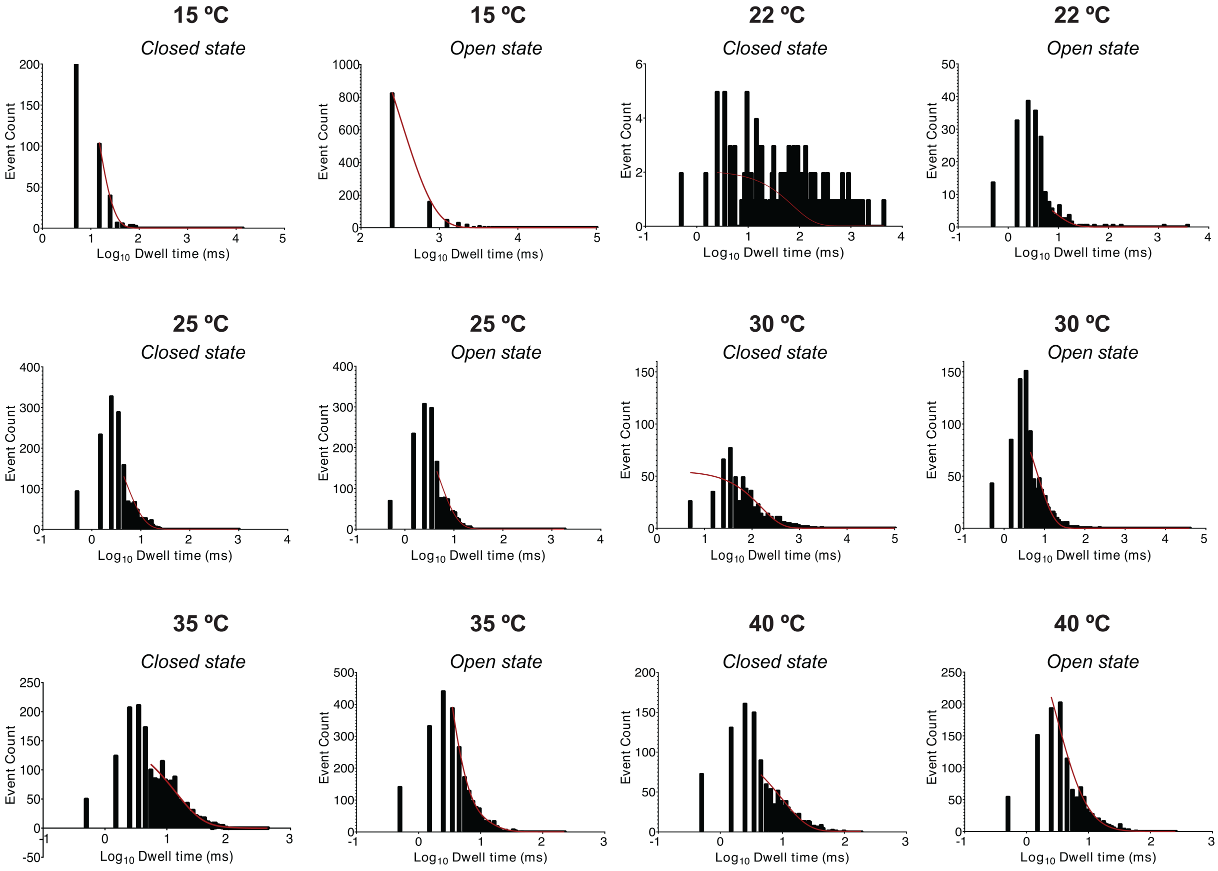

Supplementary Figure 1. Δ1-854 hTRPA1 open and closed time constants. The dwell-time histograms for Δ1-854 hTRPA1 single-channel closed and open states from corresponding experiments shown in Fig. 3. The Lorentzian distribution function was used to fit data and determine the time constant (τ) for the open and closed channel states at various indicated temperatures (see also Supplementary Table 2).

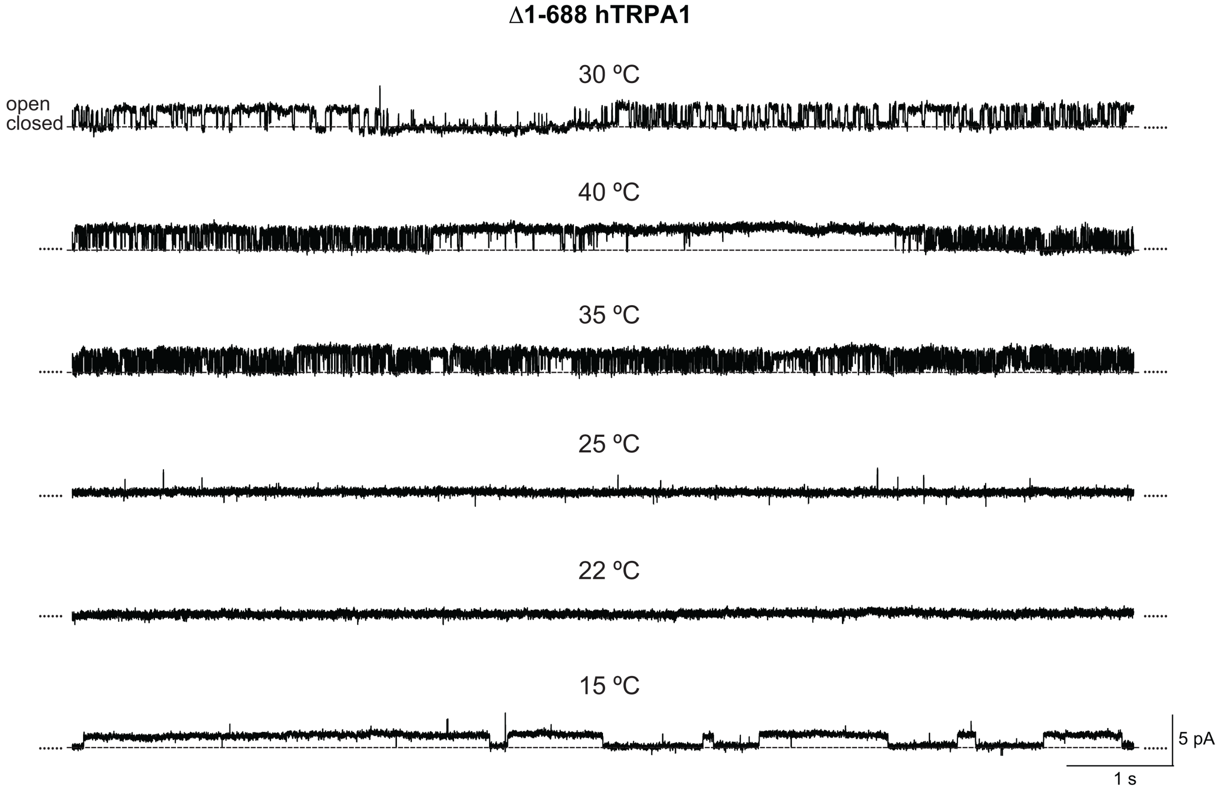

Supplementary Figure 2. The purified Δ1-688 hTRPA1 displays reversible temperature-dependent single-channel activity. Traces are part of a continuous recording (23 min) of Δ1–688 hTRPA1 single-channel currents at the indicated temperatures. The purified Δ1–688 hTRPA1 was reconstituted into planar lipid bilayers, and channel currents were recorded with the patch-clamp technique in a symmetrical K^+ solution at a holding potential of +60 mV (upward deflection shows open-channel state).

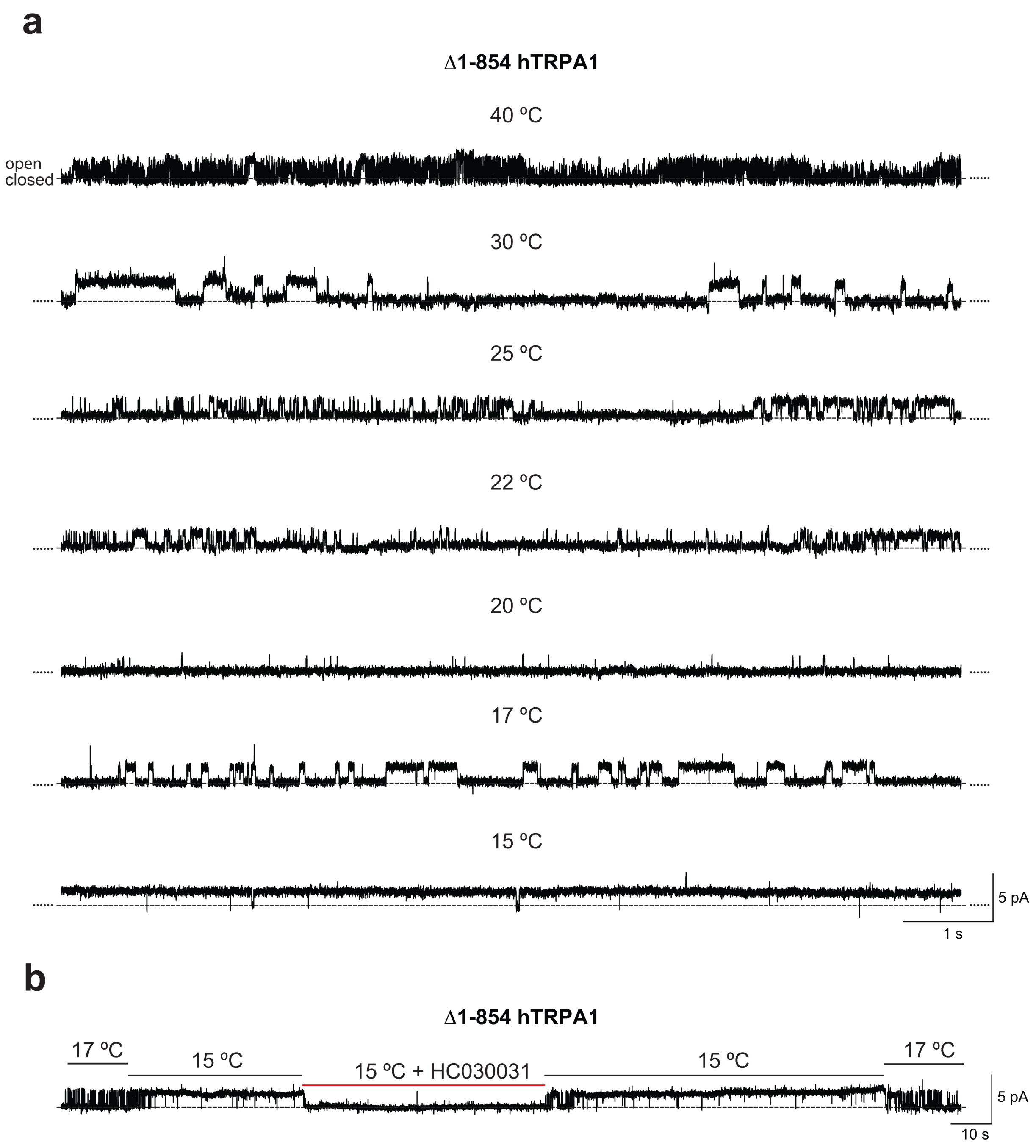

Supplementary Figure 3. The purified Δ1-854 hTRPA1 displays reversible temperature-dependent single-channel activity. a Traces are part of a continuous recording (25 min) of Δ1–854 hTRPA1 single-channel currents at the indicated temperatures. **b** As also shown in a separate recording (3.5 min total recording time), the single-cannel open probability increased dramatically by lowering the temperature within few degrees below 20°C. The inhibitory effect of the TRPA1 antagonist HC030031 (100 µM) was reversed by washout. The purified Δ1–854 hTRPA1 was reconstituted into planar lipid bilayers, and channel currents were recorded with the patch-clamp technique in a symmetrical K^+ solution at a holding potential of +60 mV (upward deflection shows open-channel state).

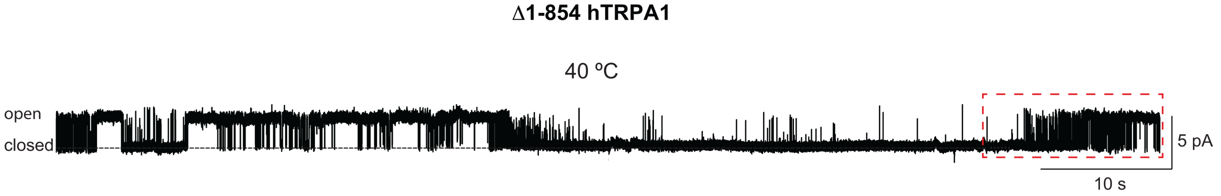

Supplementary Figure 4. The purified Δ1-854 hTRPA1 displays mixed channel behavior. The channel activity consisted of various gating patterns including interburst gaps (channel closure) of different time length and channel flickering within the same recording. Part of the recording (red dotted box) was chosen to illustrate in a single short trace (Fig. 3a) that channel activity is not monotonous over the entire recording time used for the analysis of single-channel open probability. The purified Δ1–854 hTRPA1 was reconstituted into planar lipid bilayers, and channel currents were recorded with the patch-clamp technique in a symmetrical K^+ solution at a holding potential of +60 mV (upward deflection shows open-channel state).

Supplementary Table 1. Single-channel open probability values for Δ1-688 hTRPA1 and Δ1-854 hTRPA1.

Temperature	Voltage (mV)	Δ1-688 hTRPA1 Open probability (mean ± SEM)	n	Δ1-854 hTRPA1 Open probability (mean ± SEM)	n
40 °C	+60	0.73 ± 0.09	3	0.24 ± 0.07	5
35 °C	+60	0.64 ± 0.04	9	0.17 ± 0.08	3
30 °C	+60	0.58 ± 0.04	18	0.20 ± 0.04	12
25 °C	+60	0.05 ± 0.05	8	0.25 ± 0.12	3
22 °C	+60	0.00 ± 0.00	4	0.22 ± 0.02	3
20 °C	+60	0.02 ± 0.02*	3*	0.03 ± 0.02	5
17 °C	+60	0.22 ± 0.04*	4*	0.33 ± 0.04	6
15 °C	+60	0.57 ± 0.06	5	0.92 ± 0.03	9
10 °C	+60	0.93 ± 0.02*	4*	n.d.	-

Source data are provided as a Source Data file. *Data published previously (ref. 7).

Supplementary Table 2. Time constant (τ) for Δ1-854 hTRPA1 single-channel closed and open states at various temperatures.

Temperature	Number of events (>180 s)	Closed state (ms)	Open state (ms)	P_o
40 °C	1385	86.3	23.2	0.21
35 °C	2739	51.7	18.4	0.26
30 °C	143	120	27.5	0.18
25 °C	1709	69.4	27.7	0.28
22 °C	215	61.8	10.9	0.15
20 °C	13	n.d.	n.d.	0
15 °C	1258	32	1443	0.97

Number of events were sampled for at least 180 s. The single-channel mean open probability (P_o) was calculated from time constant values, which were obtained from exponential standard fits of dwell-time histograms.
