# Supplementary material for: The human TRPA1 intrinsic cold and heat sensitivity involves separate channel structures beyond the N-ARD domain
Source: Nat Commun. 2022 Oct 17;13:6113. doi: 10.1038/s41467-022-33876-8 (PMC9576766; doi:10.1038/s41467-022-33876-8)
Supplement: Supplementary file 3 — Reporting Summary [file 41467_2022_33876_MOESM3_ESM.pdf]

nature portfolio

Corresponding author(s): Peter M Zygmunt

Last updated by author(s): September 8, 2022

Reporting Summary

Nature Portfolio wishes to improve the reproducibility of the work that we publish. This form provides structure for consistency and transparency in reporting. For further information on Nature Portfolio policies, see our Editorial Policies and the Editorial Policy Checklist.

Statistics

For all statistical analyses, confirm that the following items are present in the figure legend, table legend, main text, or Methods section.

n/a	Confirmed	
☐	☒	The exact sample size (*n*) for each experimental group/condition, given as a discrete number and unit of measurement
☐	☒	A statement on whether measurements were taken from distinct samples or whether the same sample was measured repeatedly
☐	☒	The statistical test(s) used AND whether they are one- or two-sided<br>*Only common tests should be described solely by name; describe more complex techniques in the Methods section.*
☒	☐	A description of all covariates tested
☐	☒	A description of any assumptions or corrections, such as tests of normality and adjustment for multiple comparisons
☐	☒	A full description of the statistical parameters including central tendency (e.g. means) or other basic estimates (e.g. regression coefficient) AND variation (e.g. standard deviation) or associated estimates of uncertainty (e.g. confidence intervals)
☐	☒	For null hypothesis testing, the test statistic (e.g. F, t, r) with confidence intervals, effect sizes, degrees of freedom and P value noted<br>*Give P values as exact values whenever suitable.*
☒	☐	For Bayesian analysis, information on the choice of priors and Markov chain Monte Carlo settings
☒	☐	For hierarchical and complex designs, identification of the appropriate level for tests and full reporting of outcomes
☒	☐	Estimates of effect sizes (e.g. Cohen's d, Pearson's r), indicating how they were calculated

Our web collection on statistics for biologists contains articles on many of the points above.

Software and code

Policy information about availability of computer code

Data collection	Bilayer patch-clamp data were collected using the Port-a-Patch (Nanion Technologies, Germany) and the signals were acquired with an EPC 10 amplifier (HEKA) and the data acquisition software Patchmaster (v2x 65, HEKA). Whole-cell patch-clamp data were acquired using an Axopatch 200B amplifier and the data acquisition software pCLAMP/Clampfit 9 and 10 (Molecular Devices, USA). Trp fluorescence was measured with a Cary Eclipse Fluorescence Spectrophotometer (Agilent). Circular dichroism spectroscopy was performed using Chirascan (Applied Photophysics).
Data analysis	Bilayer electrophysiological data were analysed using pCLAMP/Clampfit 9 (Molecular Devices), and Igor Pro (Wave Metrics software) and GraphPad Prism 9.1. (GraphPad Software, La Jolla, CA) were used for statistical analysis and drawing of graphs. Trp fluorescence data were analysed and visualised using GraphPad Prism 9.1. (GraphPad Software, La Jolla, CA). Whole-cell electrophysiological data were analysed using pCLAMP/Clampfit 10 (Molecular Devices, USA). SigmaPlot 10 (Systat Software Inc., San Jose, USA) was used for statistical analysis and CorelDraw X7 was used for drawing of graphs. Based on circular dichroism spectroscopy the secondary structural content was estimated using DichroWeb software (http://dichroweb.cryst.bbk.ac.uk/html/home.shtml).

For manuscripts utilizing custom algorithms or software that are central to the research but not yet described in published literature, software must be made available to editors and reviewers. We strongly encourage code deposition in a community repository (e.g. GitHub). See the Nature Portfolio guidelines for submitting code & software for further information.

Data

Policy information about availability of data

All manuscripts must include a data availability statement. This statement should provide the following information, where applicable:

- Accession codes, unique identifiers, or web links for publicly available datasets
- A description of any restrictions on data availability
- For clinical datasets or third party data, please ensure that the statement adheres to our policy

The cryo-EM structures referred to in this study are available in the Protein Data Bank under the accession codes PDB 6V9W [http://doi.org/10.2210/pdb6V9W/pdb], PDB 6V9X [http://doi.org/10.2210/pdb6V9X/pdb], PDB 6PQP [http://doi.org/10.2210/pdb6PQP/pdb]; and PDB 6V9Y [http://doi.org/10.2210/pdb6V9Y/pdb]. All electrophysiological patch-clamp data, tryptophan fluorescence data and circular dichroism spectroscopy data supporting the conclusions drawn in this study are available within the original article and/or in as supplement including Source Data file. Additional information such as raw data of these recordings are available from the corresponding authors upon reasonable request. Plasmids of the hTRPA1wild-type and mutants thereof investigated in this study are available from the corresponding authors upon request.

Field-specific reporting

Please select the one below that is the best fit for your research. If you are not sure, read the appropriate sections before making your selection.

☒ Life sciences ☐ Behavioural & social sciences ☐ Ecological, evolutionary & environmental sciences

For a reference copy of the document with all sections, see nature.com/documents/nr-reporting-summary-flat.pdf

Life sciences study design

All studies must disclose on these points even when the disclosure is negative.

Sample size	Sample size for bilayer patch-clamp and trp fluorescence assays was determined based on previous recordings using the same experimental conditions and equipment (Moparthi et al PNAS 2014, Sci Rep 2016, Cell Calcium 2020 and Moparthi & Zygmunt Cell Calcium 2020). Sample size for whole-cell patch-clamp measurements was determined based on previous experiences (Zimova et al. 2018, Sinica et al., 2019). Sample size for circular dichroism sepctroscopy studies was based on previous recordings using the same experimental conditions (Moparthi et al PNAS 2014; Survery et al JBC 2016).
Data exclusions	No data exclusions.
Replication	All attempts at replication were successful in bilayer and whole-cell patch-clamp electrophysiology studies, tryptophan fluorescence measurements as well as circular dichroism spectroscopy recordings. Bilayer patch-clamp experiments were conducted on separate bilayers and days. Whole-cell patch clamp experiments were conducted on cells from several independent transfections. Control experiments with wild-type TRPA1 were regularly conducted in order to monitor replication. Purified hTRPA1 intrinsic tryptophan fluorescence recordings were performed at different days and protein preparations. CD measurements of Δ1-854 hTRPA1 were performed at four independent occasions and each mean spectra were obtained from 25 replicate scans.
Randomization	No randomization in bilayer patch-clamp experiments as the successful insertion of TRPA1s and channel activity is a prerequisite for further analysis. As in our previous studies, protein insertion was indicated by channel activity in response to high voltage (+100 mV), intermittently tested, within 30 min. If no activity appeared the procedure was repeated with new chips and bilayers. Using this approach, the overall success rate for channel incorporation is estimated to 30%. TRPA1-mediated responses were confirmed using channel antagonists. As shown in several previous own studies, bilayers without proteins do not respond to temperature, pressure and chemicals (agonists, vehicles etc). No randomization in trp fluorescence and circular dichroism spectroscopy recordings as inherent relative changes in fluorescence and polarized light, respectively, are measured with a preset automatically run protocol and against protein free solutions. For whole-cell patch-clamp experiments, individual cells investigated are randomly chosen by the experimenter from usually > 100 transfected cells in the petri dish. Only one cell per dish is investigated. The chosen cell must be attached to the bottom of petri dish, look "healthy" and show a fluorescence indicating successful transfection.
Blinding	No blinding in bilayer patch-clamp experiments as the successful insertion of TRPA1s and channel activity is a prerequisite for further analysis. As in our previous studies, protein insertion was indicated by channel activity in response to high voltage (+100 mV), intermittently tested, within 30 min. If no activity appeared the procedure was repeated with new chips and bilayers. Using this approach, the overall success rate for channel incorporation is estimated to 30%. TRPA1-mediated responses were confirmed using channel antagonists. As shown in several previous own studies, bilayers without proteins do not respond to temperature, pressure and chemicals (agonists, vehicles etc). No blinding in trp fluorescence recordings and circular dichroism spectroscopy recordings as inherent relative changes in fluorescence and polarized light, respectively, are measured with a preset automatically run protocol and compared to protein free solutions. A true blinding in whole-cell electrophysiology was not possible, as the patch clamp experimenters also performed the transfection procedures. However, the experimenter did not know the exact nature of the mutant at the time of the experiment, this was revealed at the time of data analysis. Thus, they did not know what was predicted.

Reporting for specific materials, systems and methods

We require information from authors about some types of materials, experimental systems and methods used in many studies. Here, indicate whether each material, system or method listed is relevant to your study. If you are not sure if a list item applies to your research, read the appropriate section before selecting a response.

Materials & experimental systems

n/a	Involved in the study
	☒ Antibodies
	☒ Eukaryotic cell lines
☒	☐ Palaeontology and archaeology
☒	☐ Animals and other organisms
☒	☐ Human research participants
☒	☐ Clinical data
☒	☐ Dual use research of concern

Methods

n/a	Involved in the study
☒	☐ ChIP-seq
☒	☐ Flow cytometry
☒	☐ MRI-based neuroimaging

Antibodies

Antibodies used	Mouse Anti-6x-His Tag antibody (dilution 1:20,000; Becton Dickinson Pharmingen) and mouse monoclonal Anti-TRPA1 antibody (dilution 1:1000; Sigma Aldrich/MERCK).
Validation	Anti-TRPA1 antibody (https://www.sigmaaldrich.com/SE/en/product/sigma/wh0008989m3).<br>ouse Anti-6x-His Tag antibody (https://www.bdbiosciences.com/zh-cn/products/reagents/western-blotting-and-molecular-reagents/purified-mouse-anti-6xhis-with-control.552565).

Eukaryotic cell lines

Policy information about cell lines

Cell line source(s)	HEK293t cells (ATCC, CRL-3216, Manassas, VA, USA), Pichia pastoris (X-33, Invitrogen), High Five insect cells (BTI-Tn-5B1-4, Invitrogen) and Sf9 cells (Invitrogen).
Authentication	No authentication of any cell line used in this study.
Mycoplasma contamination	The HEK293T cells are regularly tested for mycoplasma contamination (with negative results). Cells are regularly replaced by fresh bathes free of mycoplasma. The other cell lines were not tested for mycoplasma contamination.
Commonly misidentified lines (See ICLAC register)	No commonly misidentified lines were used in this study.
